# Striking Similarities in the Presentation and Duration of Illness of Influenza A and B in the Community: A Study Based on Sentinel Surveillance Networks in France and Turkey, 2010-2012

**DOI:** 10.1371/journal.pone.0139431

**Published:** 2015-10-01

**Authors:** Jean Marie Cohen, Maria Laura Silva, Saverio Caini, Meral Ciblak, Anne Mosnier, Isabelle Daviaud, Gonçalo Matias, Selim Badur, Martine Valette, Vincent Enouf, John Paget, Douglas M. Fleming, IBGP study team

**Affiliations:** 1 Open Rome (Organize and Promote Epidemiological Network), Paris, France; 2 Réseau des GROG, Paris, France; 3 Istanbul University, Istanbul, Turkey; 4 GlaxoSmithKline (GSK) Biologicals, Brussels, Belgium; 5 Virology Department, National Influenza Center, Claude Bernard University Lyon 1, Lyon, France; 6 Virology Department, Unit of Molecular Genetics of RNA viruses, National Influenza Center, Pasteur Institute, CNRS UMR3569, Université Paris Diderot Sorbonne Paris Cité, Paris, France; 7 Netherlands Institute for Health Services research (NIVEL), Utrecht, The Netherlands; 8 Royal College of General Practitioners, Surveillance and Research Unit, Birmingham, United Kingdom; Centers for Disease Control, TAIWAN

## Abstract

Influenza B represents a high proportion of influenza cases in some seasons (even over 50%). The Influenza B study in General Practice (IBGP) is a multicenter study providing information about the clinical, demographic and socio-economic characteristics of patients affected by lab-confirmed influenza A or B. Influenza B patients and age-matched influenza A patients were recruited within the sentinel surveillance networks of France and Turkey in 2010–11 and 2011–12 seasons. Data were collected for each patient at the swab test day, after 9±2 days and, if not recovered, after 28±5 days. It was related to patient's characteristics, symptoms at presentation, vaccination status, prescriptions of antibiotics and antivirals, duration of illness, follow-up consultations in general practice or emergency room. We performed descriptive analyses and developed a multiple regression model to investigate the effect of patients and disease characteristics on the duration of illness. Overall, 774 influenza cases were included in the study: 419 influenza B cases (209 in France and 210 in Turkey) and 355 influenza A cases (205 in France and 150 in Turkey). There were no differences between influenza A and B patients in terms of clinical presentation and number of consultations with a practitioner; however, the use of antivirals was higher among influenza B patients in both countries. The average (median) reported duration of illness in the age groups 0–14 years, 15–64 years and 65+ years was 7.4 (6), 8.7 (8) and 10.5 (9) days in France, and 6.3 (6), 8.2 (7) and 9.2 (6) days in Turkey; it increased with age but did not differ by virus type; increased duration of illness was associated with antibiotics prescription. In conclusion, our findings show that influenza B infection appears not to be milder disease than influenza A infection.

## Introduction

Influenza B represents a high proportion of all cases of influenza in some seasons (even over 50%) [1, 2], and vaccine lineage inadequacy is inevitable as two antigenically distinct influenza B lineage (Victoria and Yamagata) cocirculate since 1985 with unpredictable predominant lineage [3]. Thus, there is an increasing support for the use of a quadrivalent vaccine against influenza, including both influenza B virus lineages [4].

The introduction of a vaccine in a health care system requires several studies, such as epidemiological data on the incidence, healthcare utilization, case fatality and mortality impact of the disease to be prevented in different populations and in different age groups within the same population. Evidence on comparative epidemiology and burden of disease of influenza A and B are important sources of information for the estimation of the public health impact [5] and to develop cost effectiveness analysis [6] of alternative interventions, for instance the use of quadrivalent versus trivalent influenza vaccines.

General practice based sentinel networks provide routine surveillance which are useful to develop real-time epidemiological study on several health conditions and diseases [7–9]. Regarding influenza, most sentinel networks collaborate with virology laboratories to confirm the diagnosis in suspect cases [10]. Data provided by sentinel networks have already been extensively used to plan and monitor interventions and to feed models investigating their cost effectiveness [11].

The Influenza B study in General Practice (IBGP) is, a follow up study, aiming at providing comparative information about the distribution of clinical, demographic and socio-economic characteristics in patients with a laboratory confirmed diagnosis of influenza A or B. Socio-economics results have been published elsewhere [12]. In the current paper, we present the results of a comparison of clinical presentation and duration of illness between influenza A and B cases in France and Turkey during 2010–2011 and 2011–2012 seasons.

The WHO recommendation for influenza vaccines for the northern hemisphere contained the same influenza strains for both 2010–2011 and 2011–2012 seasons: an A/California/7/2009 (H1N1)-like virus; an A/Perth/16/2009 (H3N2)-like virus; a B/Brisbane/60/2008-like virus (B/Victoria lineage) [13, 14].

## Methods

### Study design and selection of participants

The multicentre IBGP study performed a prospective recruitment of patients during two consecutive influenza seasons (from week 40 to 15): 2010–2011 (season 1) and 2011–2012 (season 2). Persons of any age with a lab confirmed diagnosis of influenza B made in the frame of established sentinel surveillance networks in France and Turkey were eligible for inclusion into the study.

The trigger for the study recruitment was the positive notification of an influenza B case by the collaborating virological laboratory. This notification was sent from the laboratory to the GP coordinators who sought to recruit the identified patient if notification occurred early enough, i.e. before eleven days after the swab specimen had been taken for investigation. If more than eleven days were elapsed from the date of swabbing, the influenza B patient was not included in the study.

For each influenza B patient included in the study, the network coordinators searched for a correspondent influenza A patient, who has been swabbed within eleven days before and belonged to the same age group. The following age groups were used: 0–4 (or, when possible, 0–2 and 3–4), 5–14, 15–49, 50–64 and 65+ years. An influenza A patient was then included according to the following algorithm:

- a patient sampled by the same GP than the influenza B patient, if possible;
- a patient sampled at the same day than the influenza B patient; or, if not possible, with the nearest date of sampling.

It was not always possible to find a suitable influenza A patient for each influenza B patient included in the study. Therefore, some influenza B patients were matched with influenza A patients that were identified later in the same season or in the following season. Selected influenza B patients were nevertheless included in the study even though a suitable influenza A patient could not be identified.

### Sentinel surveillance networks

Established sentinel surveillance networks in France and Turkey (570 and 515 physicians, respectively) [15, 16] participated in the IBGP study (S1 Appendix). The French network is nationally distributed whereas in Turkey the network is based on five provinces in the western region: Antalya, Bursa, Edirne, Istanbul and Izmir. Differently from the Turkish networks, paediatricians participate in the network in France.

The sentinel physicians monitor the incidence of influenza-like illness (ILI) and/or acute respiratory infections (ARI). In France, the GROG network adopts the wider concept of ARI defined as sudden onset of respiratory symptom(s) with infection context (fever, headache, …) in the absence of other diagnosis. In Turkey, the Ministry of Health adopts the ILI case definition of the WHO: sudden onset of fever (>38°C) with cough or sore throat, in the absence of other diagnosis. Sentinel physicians routinely investigate, by rhino-pharyngeal swabbing, a variable proportion of patients, mostly selected on an opportunistic basis, for influenza and other respiratory viruses.

Swabbed patients represent a subset of all those presenting with an ILI/ARI syndrome within two (in France) or three (in Turkey) days of symptoms onset. Due to the limited capacity of virological analysis, practitioners were asked to swab only one or two ARI/ILI patients each week. Combined nasal/throat swab specimens and a routine investigation request forms (completed for each patient) were sent by post to the respective National Influenza Centre (NIC) for virological diagnosis. In Turkey, some influenza patients were recruited in emergency departments, with direct links to the influenza laboratory; for these patients postal transport of specimens was not necessary.

The NICs followed the WHO collaborating centres recommendations for virus isolation, subtyping and lineage characterization [17]. Virus detection and subtyping of influenza A and lineage characterization of influenza B viruses were done on the samples with reverse transcriptase-polymerase chain reaction (RT-PCR), which is the most sensitive available method. Validation of the testing procedures for influenza strain typing was undertaken at the WHO Collaborating Centre for Reference and Research on Influenza of the National Institute for Medical Research (NIMR) at Hill Mill, UK, using a ten percent sample, according to the terms of reference of the NICs and WHO collaborating centres.

### Data collection

For each patient included in the study, the data collection was performed in three moments of the time horizon of the study: (1) at the swab test day (D0), (2) on recruitment to the study at 9 ± 2 days (D9), (3) in case the subject had not recovered by day 9, at 28 ± 5 days (D28).

At day 0, we used the virology investigation request form routinely used to collect data on: personal details (name, month and year of birth, gender and city code), date of onset of symptoms, presenting symptoms and syndromes, influenza vaccination status for the current season, and prescriptions of antibiotics or antivirals. Concerning vaccination status, patients were considered as vaccinated against the current seasonal flu if they had received at least one dose of seasonal vaccine in the current season and if the date of vaccination preceded by at least 14 days the onset of symptoms. Patients for which the vaccination date was unknown, were considered as not vaccinated. Information about underlying conditions was based on each country own surveillance form, which means that heterogeneous and non comparable data were collected on this topic. Data were anonymised by the lab before entry into the computer database.

**Fig 1:**
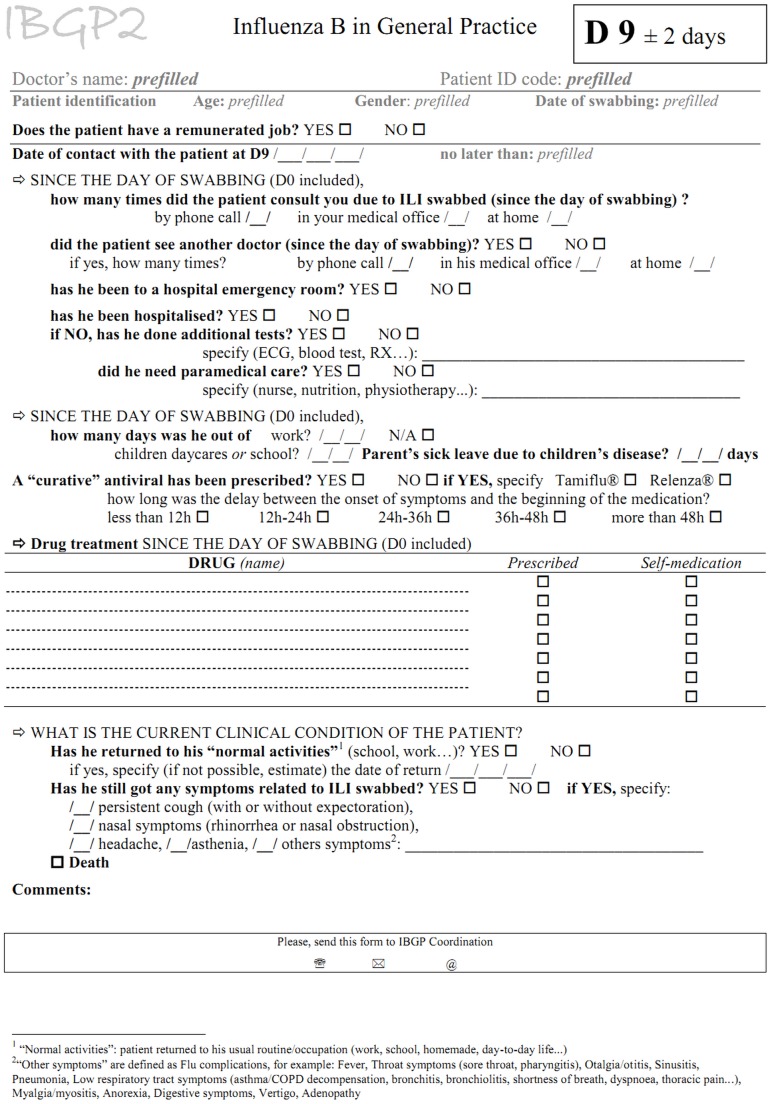
Questionnaire for data collection at days 9 and 28 in the IBGP study. France and Turkey, 2010–2012.

Further information was obtained at the time of recruitment (day 9), using a patient specific questionnaire (illustrated by an English translation in Fig 1) completed by the sentinel physician (in France) or by the study coordinator (in Turkey) on the basis of a telephone interview. Some potentially eligible influenza B patients were not recruited in France because: the lab results were not transmitted to the physician in time to contact the patient within eleven days after the swab; or the patient/GP was not reachable or did not agree to participate. Recruited patients who were not recovered at D9 were called back at D28 using the same questionnaire. Information was collected on: state of recovery (defined as date of return to normal activities), number of follow-up consultations, hospital admission and emergency room visits, drug prescriptions and details on sickness absence.

All information gathered during the IBGP study was stored electronically at the Clinical Research Organization (Open Rome headquarters, Paris).

### Analysis

Using data collected at day 0, 9 and 28, influenza A and B recruited patients were compared in terms of presenting symptoms and syndromes, vaccination status, drugs taken, duration of illness (days between symptom onset and return to normal activities), number of follow-up consultations, hospital admissions, days of absence from work or school. All comparisons were made separately for France and Turkey, and further stratified by season and age group (0–14, 15–64, 65+ years). The Fisher’s exact test was used to compare the distribution of variables of interest. A p-value was considered statistically significant when less than 0.05.

We developed a multiple regression model to investigate the effect of patients and disease characteristics on the median duration of illness including influenza type and the other variables that were significant (p<0.05) in univariate analysis. Variables considered were: influenza type, gender, age group, vaccination status, prescriptions of antibiotics and antivirals, sickness absence (defined as reported absence at follow-up interview) and season. The regression model was fitted independently for France and Turkey, and p-values were considered as statistically significant when less than 0.05. All analyses were performed with STATA software (version 11).

### Ethical approval and consent procedures

In France, ethical approval for the study (including consent procedures) was given by the concerned national ethics committee *(Comité de Protection des Personnes*, *Commission Consultative sur le Traitement de l’Information en matière de recherche dans le domaine de la santé [CCTIRS] and Commission Nationale pour l’Informatique et les Libertés [CNIL])*.

All selected influenza patients (both B and A cases) were involved in a consent procedure in two occasions: firstly in providing a swab for surveillance purpose, and then in agreeing to be included in the study (day 9 ± 2).

In France, as the IBGP study does not interfere with medical intervention, verbal informed consents were sufficient for adult participants, in accordance with the Public health code (L1122-1 à L1122-2) [18]. For recruited children, parents provided an oral consent for the D0 swabbing and, as requested by the ethics committees, a written informed consent for the D9 contact (information letter and consent form given, or read and sent by post by the practitioner at the D9 contact with a pre-stamped and preaddressed envelop for the return to the GP).

In the same way, in Turkey, in accordance with the Ministry of health recommendations on standard identification and reporting system, as the IBGP study does not interfere with medical intervention, patient or children's parents verbal informed consents are sufficient for participating and consents' record is not requested. According to the recommendation, written and separate approval for this study were not necessary as its content was present in the routine surveillance program [19].

## Results

During the two seasons, overall 4316 influenza virus detections (among which 31.9% influenza B) were identified within the two surveillance networks. Table 1 shows their distribution in terms of country, season, type, subtype or lineage. The percentage of influenza B over all influenza positive cases during season 1 and 2 was 48.2% and 3.6% in France and 53.6% and 24.2% in Turkey. Concerning influenza A, 92.7% of virus detections (seasons 1 and 2 combined) were subtyped; in both countries A(H1N1)pdm09 predominated in season 1 and A(H3N2) in season 2. Concerning influenza B, 32.0% of virus detections were characterized; the Victoria lineage predominated in France in season 1 and in Turkey in season 2, the Yamagata lineage predominated in Turkey in season 1, and the two lineages were equally frequent in France in season 2.

**Table 1:** Epidemic peak and type, subtype and lineage of influenza cases detected within the French and Turkish influenza surveillance networks during 2010–2011 and 2011–2012 seasons, and (within parenthesis, in bold) included in the IBGP study.

	2010–2011		2011–2012	
	France	Turkey	France	Turkey
**Epidemic peak (week)**	5-6/2011	5/2011	7-9/2012	2-3/2012
**Total influenza positives**	2007 **(211)**	541 **(206)**	1495 **(203)**	273 **(154)**
**Flu A (Total)**	1040 **(32)**	251 **(54)**	1441 **(173)**	207 **(96)**
AH1/pdm09	831 **(31)**	187 **(42)**	63 **(6)**	2 **(0)**
AH3	100 **(1)**	0 **(0)**	1335 **(164)**	205 **(96)**
A not subtyped	109 **(0)**	64 **(12)**	43 **(3)**	0 **(0)**
**Flu B (Total)**	967 **(179)**	290 **(152)**	54 **(30)**	66 **(58)**
Victoria	274 **(155)**	0 **(0)**	16 **(15)**	53 **(51)**
Yamagata	18 **(11)**	60 **(58)**	19 **(14)**	0 **(0)**
B not characterized	675 **(13)**	230 **(94)**	19 **(1)**	13 **(7)**

A total of 774 patients were included in the study: 355 influenza A (205 in France and 150 in Turkey) and 419 influenza B (209 in France and 210 in Turkey) (Table 1). Males represented 53% and 49% of influenza recruits in France and Turkey, respectively. The proportion of influenza B cases aged 0–14, 15–64 and 65+ years was 67%, 27% and 6% in France and 27%, 67% and 6% in Turkey (Table 2); the age distribution of influenza A cases mirrors that of B patients, due to matching.

**Table 2:** Gender, age groups, presenting symptoms and syndromes, hospitalization, vaccination status and prescriptions of antibiotics and antivirals among patients enrolled in the IBGP study, by country and influenza type. France and Turkey, 2010–2012.

	France			Turkey		
	Influenza A (n = 205)	Influenza B (n = 209)	P	Influenza A (n = 150)	Influenza B (n = 210)	P
**Gender**						
male	55%	50%	ns	51%	48%	ns
**Age groups (years)**						
0–14	67%	67%		29%	27%	
15–64	27%	27%	ns	66%	67%	ns
65+	6%	6%		5%	6%	
**Presenting symptoms and syndromes**						
fever	99%	100%	ns	87%	89%	ns
cough	90%	92%	ns	91%	95%	ns
rhinitis	71%	71%	ns	87%	81%	ns
headache	66%	65%	ns	78%	74%	ns
myalgia	60%	63%	ns	78%	78%	ns
sore throat	56%	50%	ns	45%	36%	ns
dyspnea	5%	6%	ns	7%	12%	ns
**Hospitalization**						
yes	1%	1%	ns	3%	13%	0.002
**Vaccination status**						
vaccinated	5%	6%	ns	5%	1%	0.028
**Drugs prescribed**						
antibiotics	28%	24%	ns	51%	58%	ns
antivirals	13%	21%	0.031	15%	31%	0.001

ns: not significant

In France, the profile of symptoms at presentation was similar among influenza A and B recruits, with fever and cough being present each in >75% of patients. In Turkey, most frequent symptoms were cough, fever, rhinitis, myalgia and headache. Very few (1%) of recruited subjects were hospitalized in France, (no differences by influenza type) while this percentage was 9% in Turkey (13% of influenza B recruits vs 3% influenza A, p<0.01). The proportion of recruits that were vaccinated against influenza was 5–6% in France.

In Turkey, influenza A recruits were frequently more vaccinated than influenza B recruits (p<0.03). Antibiotics were prescribed to 26% of recruits in France and to 55% in Turkey (no differences by influenza type). Antivirals were prescribed more frequently for influenza B than for influenza A recruited patients in Turkey (respectively 15% vs 31%, p = 0.001) and in France (respectively 13% vs 21%, p<0.05). In particular, the use of antiviral was higher for B than A influenza patients in Turkey only among those aged 15–64 years (32% vs. 20%, p <0.05) and 65+ years (85% vs. 14%, p<0.01). Proportion of missing data was low (below 2%) for all variables.

Most influenza recruited patients consulted a physician only once (Table 3). The mean number of consultations was similar for influenza A and B recruits in Turkey (respectively 1.08 and 1.04) and France (respectively 1.39 and 1.38), except among influenza B recruits aged 15–64 years in France which tend to consult more frequently (p<0.01). The number of consultations increased with age in both influenza A and B recruited patients in France (p<0.01) but not in Turkey.

**Table 3:** Number of consultations with practitioners of patients infected with influenza B and A, by age groups. France and Turkey, 2010–2012.

	France				Turkey			
**Influenza A cases**								
	0–14 yrs (n = 138)	15–64 yrs (n = 55)	≥ 65 yrs (n = 12)	all (n = 205)	0–14 yrs (n = 44)	15–64 yrs (n = 99)	≥ 65 yrs (n = 7)	all (n = 150)
Mean No. of consultations	1.36	1.35	2.00	1.39	1.10	1.07	1.14	1.08
No. of consultations (%)								
once	74%	69%	17%	69%	91%	94%	86%	92%
twice	20%	27%	66%	25%	9%	5%	14%	7%
three or more times	6%	4%	17%	6%	0%	1%	0%	1%
**Influenza B cases**								
	0–14 yrs (n = 139)	15–64 yrs (n = 57)	≥ 65 yrs (n = 13)	all (n = 209)	0–14 yrs (n = 57)	15–64 yrs (n = 140)	≥ 65 yrs (n = 13)	all (n = 210)
Mean No. of consultations	1.21	1.68	1.92	1.38	1.09	1.02	1.00	1.04
No. of consultations (%)								
once	84%	47%	46%	72%	93%	98%	100%	97%
twice	12%	39%	15%	20%	5%	1%	0%	2%
three or more times	4%	14%	39%	8%	2%	1%	0%	1%

The average (median) reported duration of illness in the age groups 0–14, 15–64 and 65+ years was 7.4 (6), 8.7 (8) and 10.5 (9) days in France, and 6.3 (6), 8.2 (7) and 9.2 (6) days in Turkey, with no differences by influenza type. The results of the uni- and multi-variable regression examining the impact of the variables considered on the duration of illness are given in Table 4. In multivariable analysis, influenza type does not significantly modify the duration of illness in either country. In both France and Turkey, the fact to be younger than 15 years (compared to those aged ≥15) was significantly associated with a reduced duration of illness while an increased duration of illness was significantly associated with having been prescribed antibiotics. In Turkey, an increased duration of illness was significantly associated with having flu during 2010–11 season in univariate, but not multiple regression analysis.

**Table 4:** Uni- and multi-variable regression analysis of variables impacting on duration of illness. France and Turkey, 2010–2012.

	Univariate analysis	Logistic multi-variable analysis			
Covariate	Kolmogorov-Smirnov p-value	Odd Ratio	95% CI		p-value
**France**					
Male gender	0.137				
Age <15 years	**0.000**	**0.56**	**0.37**	**0.85**	**0.007**
Age ≥ 65 years	0.062				
Influenza B	0.348				
Season 2010–11	0.261				
Vaccination against influenza	0.678				
Antivirals prescribed	0.711				
Antibiotics prescribed	**0.000**	**2.19**	**1.39**	**3.45**	**0.001**
Sickness absence	0.469				
**Turkey**					
Male gender	0.531				
Age <15 years	**0.005**	**0.43**	**0.27**	**0.71**	**0.001**
Age ≥ 65 years	0.459				
Influenza B	0.423				
Season 2010–11	**0.000**	0.76	0.49	1.20	0.241
Vaccination against influenza	0.559				
Antivirals prescribed	0.537				
Antibiotics prescribed	**0.006**	**2.12**	**1.38**	**3.27**	**0.001**
Sickness absence	0.204				

## Discussion

We successfully recruited 419 influenza B and 355 influenza A laboratory confirmed cases providing a database in which we would expect to detect important differences (if any) between influenza A and B presentation. We compared the main characteristics of influenza in two countries (France and Turkey) during two consecutive influenza seasons (2010–11 and 2011–12). To the best of our knowledge, this is the largest European community-based study that compares influenza A and B in recent years. This was made possible by the fact that influenza B circulated intensely in Europe during the 2010–2011 season [20].

We found no significant differences between influenza A and B recruited patients for most of the aspects we considered, including presenting symptoms and syndromes, number of consultations and duration of illness. We found more hospital/emergency room visits for influenza B in Turkey though these may have been influenced by changes in healthcare access in Turkey between the two seasons. The majority of influenza B and A cases in Turkey were respectively enrolled during seasons 1 and 2, i.e. before and after the Turkish healthcare reform. Before the reform in 2011, a large proportion of the population did not have any health insurance; however, free healthcare was provided in hospitals, encouraging ill people to primarily search for hospitals. Therefore, these influenza patients were registered as hospitalized patients. After the 2011 reform, there was a stricter control on direct access of patients to the emergency rooms of hospitals.

As shown by Irving et al. [21], no consistent difference exists in the course of disease for patients affected by either influenza A or B. Other papers have focused on the paediatric population [22–25], hospitalized patients [26, 27], or other specific populations [28, 29]. No consistent patterns emerged that would help differentiate between influenza A and B patients solely on the basis of their clinical presentation, except for milder symptoms among pandemic A(H1N1) than A(H3N2) patients.

This study highlights an increased duration of illness significantly associated with having been prescribed antibiotics, in both France and Turkey, probably because antibiotics are usually prescribed when influenza clinical pattern is severer or followed by bacterial infections. Campaigns against inappropriate prescription of antibiotics in patients with ARI were conducted in France but not in Turkey in recent years; thus the higher proportion of antibiotics prescription in the latter was not surprising. Likewise, health authorities in France published recommendations for antivirals prescription, specifying that it should be only given to ARI patients characterized as clinically severe (i.e. with complications) or having specific risk factors (i.e. diabetes) [30]. Antivirals were more frequently prescribed in influenza B cases compared to A cases in our study. This could be explained by the fact that influenza B cases were mostly recruited during 2010–2011 season (post pandemic season with intense circulation of A(H1N1)pdm09) whereas influenza A cases were mostly recruited during 2011–2012 season.

The low proportion of patients that were vaccinated against influenza in our study sample may be partly explained by the low coverage rate of healthy children and adults in France and Turkey [31, 32] and by the effectiveness, although not perfect, of the influenza vaccination among the elderly. Both in France and Turkey, an increased duration of illness is described among elderly patients, emphasizing that they are more exposed to more severe clinical patterns.

The limitations of our study are mainly due to sentinel surveillance peculiarities. There were inevitable delays in the surveillance program to get the virological result, including the delays between illness onset and consultation in which the patient is swabbed; postal transit of swab test/form from medical office to laboratory; completion of virological investigation and transmission of result to sentinel physician; physician receipt of result and effective telephone contact with eligible patient within the recruitment time window. These delays strongly limited eligible patients’ recruitment to the follow-up study.

Sampling or virological analysis bias may occur. However, it is not part of the routine surveillance to investigate every ILI/ARI patient, because of work load and costs constraints that limit the laboratory investigation. In the same way, the virological investigation would ideally include strain subtyping and lineage characterization of all samples but these are not undertaken routinely. As influenza A and B cases were matched by age, we could not compare the age distribution between the two influenza types. The limitations on recruitment to follow-up limited the opportunity for meaningful secondary analyses such as separate analysis by co-morbidity. The discretion left to participating practitioners in sampling the ARI/ILI patients may introduce a sampling bias and distort some results, for instance those on the clinical presentation and the proportion of vaccinated. Recall bias may be also an issue, as some information was collected long time after the onset of symptoms.

Differences in access to healthcare between France and Turkey especially concerning the care of children limited opportunities for comparing the results in the two countries. The national differences and distinct arrangements for routine surveillance limited the use of pooled analyses where less objective clinical outcome diagnoses are compared. These practical limitations need to be addressed when planning similar studies.

In conclusion, we showed in two distinct settings that the presentation and the duration of illness due to influenza B do not differ from influenza A. Influenza B is not a milder disease than influenza A and, although it is (on average) less frequent than the latter, the efforts aiming at reducing its burden of disease should be as great as possible. Several differences were instead observed between influenza patients in France and Turkey, which most likely depend on the large dissimilarities in the healthcare systems between these two countries. This raises doubts about the comparability of studies that, unlike the IBGP, are conducted in different countries applying differently the same study protocol.

## Supporting Information

S1 Appendix Influenza surveillance network in France and Turkey. (DOCX)
