# Supplementary material for: Striking Similarities in the Presentation and Duration of Illness of Influenza A and B in the Community: A Study Based on Sentinel Surveillance Networks in France and Turkey, 2010-2012
Source: PLoS One. 2015 Oct 1;10(10):e0139431. doi: 10.1371/journal.pone.0139431 (PMC4591015; doi:10.1371/journal.pone.0139431)
Supplement: S1 Appendix — (DOCX) [file pone.0139431.s001.docx]

***S1 Appendix: Influenza surveillance network in France and Turkey***

## Influenza surveillance network by country

The influenza surveillance in each European country follows the recommendations provided by WHO and EISN. Some information and numbers for the networks regarding the present project is following given.

### France – GROG network

The establishment of the Regional Groups for Flu Observation Network – GROG – was based on the fact that doctors and primary caregivers were in the "front line" facing the influenza epidemic.

Founded in 1984, the GROG network, a network of early warning for influenza, has become a symbol of the participation of general practitioners into public health.

The GROG network participates as a correspondent in the National Health Service, since 2004, and covers 21 of the 22 metropolitan areas^[[1]](#footnote-1)^. GROG sentinel practitioners are specifically trained by the national coordination and have the equipment which enables them to take samples in search of respiratory infectious agents. The specimens are collected in general population based on a weekly comparison of linked clinical and virological data compilation. Detection of influenza viruses is performed in rhinopharyngeal samples taken in community practices and mostly tested in laboratories. Recommendations are to swab within the first 2 days after onset of the symptoms, throughout nasal swabs or throat swabs.

The laboratories provide the kit for swabbing (ViroCult or UTM Copan). The specimens collected are sent to laboratories by post. All the virological samples sent to laboratories for analysis are accompanied with a clinical description.

A feedback is provided to these sentinel practitioners and it is a valuable tool for dissemination of warning signals to the clinicians and the public. Additionally, the regionalized structure helps maintain throughout France, a network proximity, request and adaptable when necessary.

**GROG network in numbers**

The total of sentinels for the season 2012/2013 are 526, being 411 GPs and 115 paediatricians.

French network 2011/2012 – 2012/2013

| **Specimen 2011/2012** | | | **Specimen 2012/2013** | | |
| --- | --- | --- | --- | --- | --- |
| Flu negative | Influenza A | Influenza B | Flu negative | Influenza A | Influenza B |
| 2924 | 1370 | 47 | 2884 | 1120 | 1370 |
| **4341** | | | **5374** | | |

**GROG network staff responsible for the study**

- Anne Mosnier (network surveillance coordination)
- Isabelle Daviaud (data management)
- Tai Tan Bui (informatics)
- Sylvie Van der Werf (virological laboratory coordination – North France)
- Bruno Lina (virological laboratory coordination – South France)

### Turkey – Istanbul Influenza Center

Before 2003, there was no information from Turkey regarding flu activity on FluNet from the WHO. In 2003-2004 season a small scale surveillance launched by Istanbul Faculty of Medicine generated the first available influenza information in Turkey.

Then in 2005-2006 season, sentinel influenza surveillance was launched by MoH which was to be conducted by Istanbul Faculty of Medicine Laboratory and by National Hygiene Center in Ankara. Currently, sentinel surveillance is conducted in 14 major provinces and National Influenza Reference Laboratory at Istanbul Faculty of Medicine in Istanbul receives samples from five major cities in the western part of the country; whereas, MoH receives samples from the rest of the 9 selected cities from the other parts of the country.

The laboratory visits the sentinels of each city they receive samples for analysis. These sentinels are then trained about the importance of the quality of the specimen collected (at the right time, from the right place and in adequate amount), its storage and transport. The recommendations are to swab within the first 3 days after onset of the symptoms, throughout nasal swabs or throat swabs. The laboratories provide the kit for swabbing (ViroCult) and Transportation (Cargo).

The sentinels are encouraged to complete a form which allows the laboratory to determine the percentage of ILI cases among weekly visitors of a polyclinic. The patient information form enables identifying whether the patient has underlying disease, vaccinated, given antivirals, etc. During an annual surveillance, there is approximately 600-1000 samples transported and analyzed. Samples are directly sent to the laboratories along with patient information forms

**Istanbul Influenza Center in numbers**

The total of sentinels for the season 2009/2010 were 515, being 500 GPs and 15 paediatricians.

**Istanbul network staff responsible for the study**

- Meral Ciblak (coordination and data management)
- Selim Badur (coordination)

1. In Corsica, the GROG is not yet fully structured despite the presence of several GPs cooperating with this network. [↑](#footnote-ref-1)
